# Comparative Therapeutic Efficacy of ^153^Sm-EDTMP and ^177^Lu-EDTMP for Bone Pain Palliation in Patients with Skeletal Metastases: Patients’ Pain Score Analysis and Personalized Dosimetry

**DOI:** 10.3389/fmed.2017.00046

**Published:** 2017-05-01

**Authors:** Sarika Sharma, Baljinder Singh, Ashwani Koul, Bhagwant Rai Mittal

**Affiliations:** ^1^Department of Nuclear Medicine and PET, Postgraduate Institute of Medical Education and Research, Chandigarh, India; ^2^Department of Biophysics, Panjab University, Chandigarh, India

**Keywords:** ^153^Sm-EDTMP, ^177^Lu-EDTMP, prostate/breast cancer, skeletal metastases, bone pain palliation, pain scoring, patients’ dosimetry

## Abstract

**Introduction:**

The aim of the present study was to compare the therapeutic efficacy of ^153^Sm-EDTMP and ^177^Lu-EDTMP in pain palliation in cancer patients with skeletal metastases.

**Materials and methods:**

Thirty patients (25 M:5 F, mean age: 66.0 ± 14.7 years) of breast/prostate cancer with documented skeletal metastases were recruited prospectively. Twenty patients were considered randomly for treatment with ^153^Sm-EDTMP and with ^177^Lu-EDTMP in 10 patients, respectively. Using fixed dose of 37.0 MBq/kg body weight of each, the mean administered doses of ^153^Sm-EDTMP and ^177^Lu-EDTMP were 2,155.2 ± 419.6 MBq (1,347–2,857) and 1,935.1 ± 559.4 MBq (1,073–2,627), respectively. Anterior and posterior whole body images were acquired at different time points following radioactivity administration. The first data set of pre-void images (acquired at 0.5 h) representing the total activity of either of ^153^Sm-EDTMP or ^177^Lu-EDTMP was considered as reference images. All the serial images were used for patients’ dosimetry analysis by using organ level internal dosimetry assessment algorithm. Reduction in pain scoring was assessed clinically over 8 weeks by using appropriate WHO criteria and correlated with the absorbed dose to the metastatic sites.

**Results:**

A total of 86 metastatic lesions clearly visualized on post-therapy serial images (matching on bone scans) were evaluated for absorbed dose calculations. Both ^153^Sm-EDTMP and ^177^Lu-EDTMP delivered similar absorbed dose to the metastatic sites, i.e., 6.22 ± 4.21 and 6.92 ± 3.92 mSv/MBq, respectively. The mean absorbed doses to various other organs were found to be comparable and within the safe limits. A complete response (CR) for each radionuclide was evaluated as 80.0%. No significant alternation in blood parameters and no untoward reaction were observed. However, a mild to severe toxicity was observed in two patients (1 each with ^153^Sm-EDTMP and ^177^Lu-EDTMP). Kaplan–Meier survival analysis demonstrated that 27/30 patients had pain-free survival (CR) up to the observational period of 8 weeks. However, no statistically significant correlation could be established between the pain scoring and absorbed dose to metastatic sites.

**Conclusion:**

Both the radionuclides thus offer an effective and comparable therapeutic efficacy for bone pain palliation at an affordable cost and can be used interchangeably as per the availability.

## Introduction

Skeletal metastases remain a major cause of morbidity and mortality in 65–75% of the patients with advanced breast and prostate cancer (1–3). The consequences of bone metastases include pathologic fractures, life-threatening hypercalcemia, spinal cord compression, and other nerve-compression complications associated with severe and persisting pain. Often, the management of pain due to wide spread skeletal metastases is not only a challenge to the treating oncologists but also adds to the financial and social burden on the family of such patients (4–6).

The palliative care does not provide any survival benefits but it improves the quality of life by pain reduction (7). The spectrum of palliative treatment ranges from non-steroidal analgesics to opioids, chemotherapy or hormonal therapy, as well as radiation treatment using external beam irradiation or systemic radionuclide therapy (6, 8–11). However, after the initial standard palliative treatment, about 50% of these patients still continue to have substantial bone pain (12). Pain due to bone metastases is usually the first clinical symptom of the disease which increases in severity with advancing disease stage and duration (13). Therefore, bone pain palliation requires appropriate therapies for improving the quality of life in these patients (14, 15).

Radionuclide therapy has the advantage of targeting all the involved osseous sites simultaneously. The selective absorption/uptake of these bone seeking therapeutic radio pharmaceuticals provides high target to non-target (T/NT) ratio to achieve best possible palliation effects (9, 16). Multidentate polyaminophosphonic acids have been demonstrated as potential molecules for labeling with radiolanthanides and with other +3 metal ions for developing agents suitable for bone pain palliation (17). ^153^Sm-EDTMP which localizes preferentially in areas of increased osteoblastic activity has been approved by FDA for bone pain palliation secondary to metastases (18). It possesses ideal physical properties, i.e., medium-energy β-particles ranging between 640 and 810 keV limiting the tissue range to 3.0 mm, half-life of 1.95 days and has ɤ-emission of 103 keV which permits imaging of its skeletal distribution with conventional gamma cameras. Also, ^177^Lu-EDTMP is considered as an excellent radionuclide for bone pain palliation owing to its favorable physical (*T*_1/2_ = 6.73 days; *E*β_max_ = 497 keV; *E*_ɤ_ = 113, 208 keV) characteristics suitable both for treatment and scintigraphic localization of the bone metastatic sites. And an indigenous large-scale production of both ^177^Lu and ^153^Sm in adequate specific activities is feasible in India using moderate flux research reactor to provide treatment to the patients at an affordable cost (19–21).

In the present study, we evaluated the comparative therapeutic efficacy of ^153^Sm-EDTMP and ^177^Lu-EDTMP for bone pain palliation in prostate and breast cancer patients with multiple skeletal metastases. The absorbed dose to the metastatic lesions was evaluated using organ level internal dosimetry assessment (OLINDA) approach (22, 23). The outcome of the therapeutic efficacy of both the therapeutic radionuclides was measured as a function of decrease in pain following appropriate clinical criteria for pain assessment.

## Materials and Methods

### Patients’ Selection

Thirty patients (25 M:5 F, mean age: 66.0 ± 14.7 years) of breast/prostate cancer with documented skeletal metastases were recruited during the study period (January, 2012–January, 2015) prospectively. The patients were randomly divided into two groups. The first group (*n* = 20) was considered for radionuclide therapy with ^153^Sm-EDTMP and the second group (*n* = 10) with ^177^Lu-EDTMP. The patients were kept blinded to the treatment received. Prior to administration of radionuclide, each patient underwent a detailed history, clinical examination, ^99m^Tc-methylelene diphosponate (^99m^Tc-MDP) bone scanning, and various blood investigations.

Only the patients with positive ^99m^Tc-MDP bone scanning (within the last 8 weeks) as evidence of multiple skeletal metastases, having severe bone pain despite receiving analgesics, not candidates for local external beam radiation therapy and who had given a written and informed consent were included in the study. The other inclusion criteria were patients not having received any chemotherapy or external beam therapy during the last 4–12 weeks and with normal hematological/renal parameters. And patients with absolute contraindications for pregnancy/lactation, pre-existing cytopenia, super “bone scan appearance” and having any previous documented history of hypersensitivity or reaction to radionuclide/radiopharmaceutical administration were excluded from the study.

### ^99m^Tc-MDP Bone Scanning

A whole body (anterior and posterior) bone scan was performed in all the patients using dual headed gamma camera at 3.0 h following intravenous administration of about 555.0–740.0 MBq of ^99m^Tc-MDP.

### Radionuclide Treatment and Whole Body Scintigraphy

^153^Sm-EDTMP and ^177^Lu-EDTMP were procured as multiple consignments (Board of Radiation Isotope and Technology, Mumbai, India). Prior to use, radiolabeled product from each consignment was subjected to routine quality checks. The patients were administered intravenously either with ^153^Sm-EDTMP or ^177^Lu-EDTMP at a dose rate of 37.0 MBq/kg body weight. The safety of the radionuclide treatment was assessed using the Common Terminology Criteria for Adverse Events criteria, version 4.0 (24). All the patients were treated on an Out Patient Department basis.

Imaging was performed by using two gamma cameras (either Symbia T-16, Siemens, Erlangen, Germany or Infinia Hawkeye-4, GE, Milwaukee, WI, USA) fitted with low-energy high resolution collimator. Each gamma camera was peaked to the energy of the respective radionuclide. The sensitivity or calibration factor (factor for converting counts per minute per cm to MBq) for each gamma camera and the dose calibrators used in the study were calculated for both the radionuclides by using a standard method (25, 26). This calibration factor was used for patients’ dosimetry calculations.

The patients lied in supine position on the imaging table. The whole body (anterior and posterior) images were acquired in 1,024 × 256 matrix at a scan speed of 10.0 cm/min. The imaging in each patient was performed at 0.5 h, 3 h, 6 h, 24 h, 48 h, 96 h, and 5–6 days post radioactivity administration. The first data set of anterior and posterior images acquired at 0.5 h (without allowing the patients to void) represented 100.0% of the administered activity of either of ^153^Sm-EDTMP or ^177^Lu-EDTMP. This data set of images was considered as reference images for patients’ dosimetry analysis.

### Data Analysis

All the lesions/organs which were clearly visualized (and had higher tracer uptake than the background) on 0.5 h whole body anterior and posterior reference images were identified. A region of interest (ROI) on each of the identified lesion/organ was drawn on the reference 0.5 h whole body anterior and posterior images. Exactly the same sized ROIs were then replicated on the corresponding regions on the serial delayed images of each patient. The background corrected counts for each organ were divided by the number of pixels within the ROI. In each patient, a total of seven regions (whole body, brain, bladder, right kidney, left kidney, thigh muscles, and normal femur bone) each on anterior and posterior images were included for this semi-quantitative analysis.

The geometric means of the background corrected counts/pixel calculated for each lesion/organ were converted into percent fraction of the total injected activity. The following formula was used to calculate the %ID:

$$\%{\mathrm{ID}}_{\text{uncorrected}}=\frac{C_{\text{ROI}/\text{pixel}}}{C_{\text{WB}/\text{pixel}}}\times 100$$

where % ID_uncorrected_ = uncorrected percentage of injected activity (later corrected for decay factor), C_ROI/pixel_ = counts per pixel in the ROI, and C_WB/pixel_ = counts per pixel in the whole body ROI.

The % ID values calculated for various regions including the skeletal metastatic sites on the serial set of anterior and posterior whole body images were analyzed using the OLINDA software (version 1.0) for absorbed dose estimates. The %ID values were plotted against time for each organ. Area under the curve analysis of the time activity curve of an organ/lesion represented the number of disintegrations or cumulative activity of the representative organ. The number of disintegrations for the source organ was obtained using the OLINDA/EXM kinetic input model, applying a mono or bi-exponential fit to the data of each source region/organ. The radiation absorbed dose in mSv/MBq in the target organs and the metastatic lesions was estimated by inserting the corresponding number of disintegrations and dose factors of source organ (derived from OLINDA) for each of the organs. Residence time was calculated by dividing number of disintegrations with injected activity. The whole body effective dose and effective dose equivalents were also evaluated by this data analysis.

### Pain Relief Assessment Following Radionuclide Therapy

The therapeutic efficacy of each of the two radionuclides at post-therapy periods of 1, 3, 6, and 8 weeks was evaluated by using standard pain scoring assessment criteria (8). Based upon this assessment, the response was labeled as (a) complete response when the pain score was <3.0, (b) partial response when the pain score ranged between 4 and 8, and (c) no response when the pain score was >8.0 and had no change from the baseline score.

### Statistical Analysis

The quantitative data evaluated as mean ± SD for the mean absorbed dose (for different lesions/organs) obtained for two different radiopharmaceuticals in two groups of patients was compared using the independent student “*t*” test. The pain scores among responders and non-responders within the two groups were compared using paired Student “*t*” test. The *p* value of <0.05 was considered significant for all tests at 95% confidence interval. The data were analyzed using Statistical Package for the Social Sciences (27).

## Results

The mean administered doses of ^153^Sm-EDTMP and ^177^Lu-EDTMP did not differ significantly (as we used 37.0 MBq/Kg body weight for both the radiopharmaceuticals) and were 2,155.2 ± 419.6 (range 1,347–2,857) MBq and 1,935.1 ± 559.4 (range 1,073–2,627) MBq, respectively. No significant alternation in the blood parameters was observed (in comparison with the baseline values) at post-therapy follow-up periods of 1, 3, 6, and 8 weeks with either of the two radiopharmaceuticals (Tables 1 and 2).

**Table 1:** **Baseline laboratory parameters (mean ± SD) of patients treated with ^153^Sm-EDTMP**.

Patient investigations	Laboratory values at different time intervals				
	Pretherapy	1 week	3 weeks	6 weeks	8 weeks
Hemoglobin (g/dL)	9.94 ± 0.59	9.4 ± 0.80	8.9 ± 0.89	8.5 ± 2.11	8.8 ± 2.15
Absolute WBC counts (/mm^3^ of blood)	8,930 ± 1,290	6,335 ± 1,311	6,285 ± 1,491	6,145 ± 1,759	6,775 ± 1,754
Neutrophil counts (/mm^3^ of blood)	5,542 ± 1,172	2,336 ± 550	2,019 ± 391	1,800 ± 557	2,164 ± 618
Platelet counts (/mm^3^ of blood)	2.50 ± 0.42	1.74 ± 0.32	1.13 ± 0.29	0.91 ± 0.25	1.31 ± 0.46

**Table 2:** **Baseline laboratory parameters (Mean ± SD) of patients treated with ^177^Lu-EDTMP**.

Patients investigations	Laboratory values at different time intervals				
	Pretherapy	1 week	3 weeks	6 weeks	8 weeks
Hemoglobin (g/dL)	9.7 ± 0.33	8.4 ± 0.42	8.6 ± 0.42	8.5 ± 0.49	8.8 ± 0.46
Absolute WBC counts (/mm^3^ of blood)	8,630 ± 988	5,590 ± 1,278	5,460 ± 1,322	5,840 ± 818	7,240 ± 834
Neurophil counts (/mm^3^ of blood)	5,261 ± 647	1,888 ± 434	2,101 ± 311	2,319 ± 636	2,466 ± 352
Platelet counts (/mm^3^ of blood)	2.14 ± 0.25	1.53 ± 0.28	1.10 ± 0.21	0.98 ± 0.17	1.38 ± 0.38

**Figure 1:**
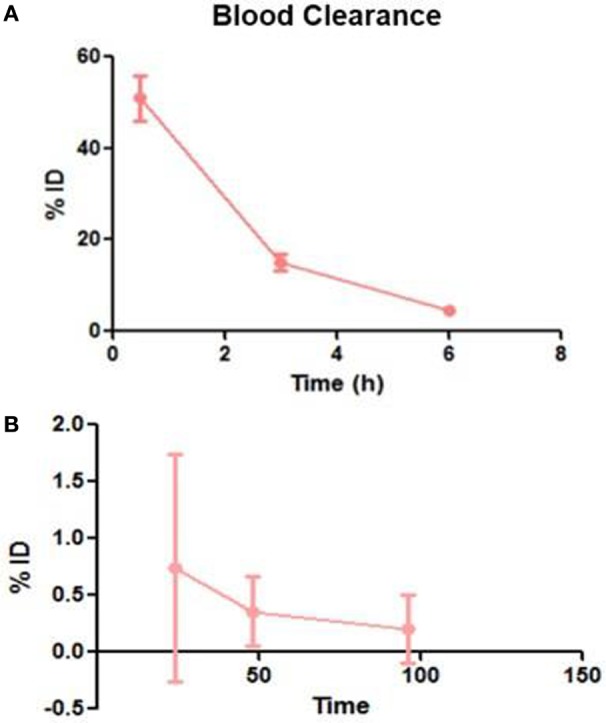
**Blood clearance pattern of ^153^Sm-EDTMP**. **(A)** Percent fraction of the injected dose (%ID) remaining in blood over the first 6 h **(B)** over the next 96 h.

**Figure 2:**
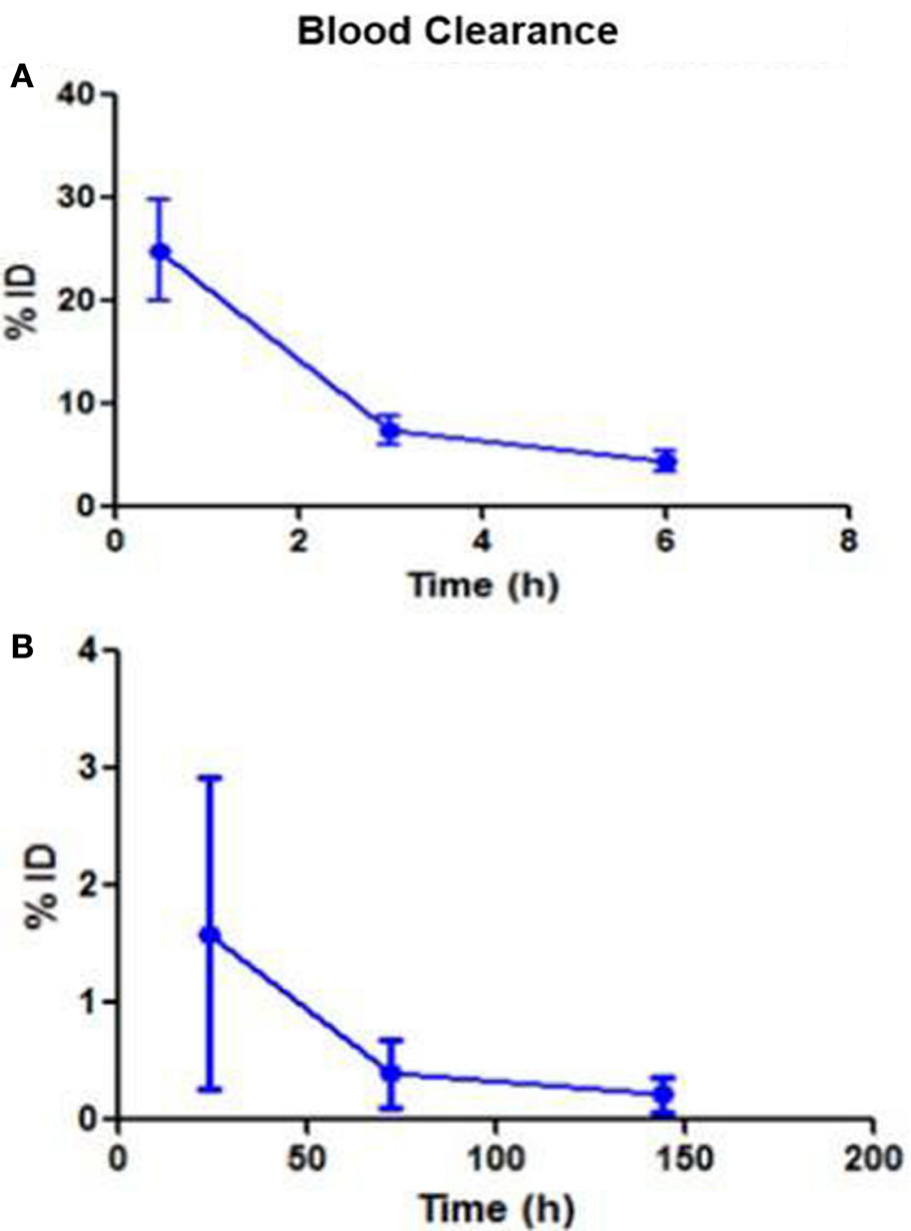
**Blood clearance pattern of ^177^Lu-EDTMP**. **(A)** Percent fraction of the injected dose (%ID) remaining in blood over the first 6 h **(B)** over the next 144 h.

**Figure 3:**
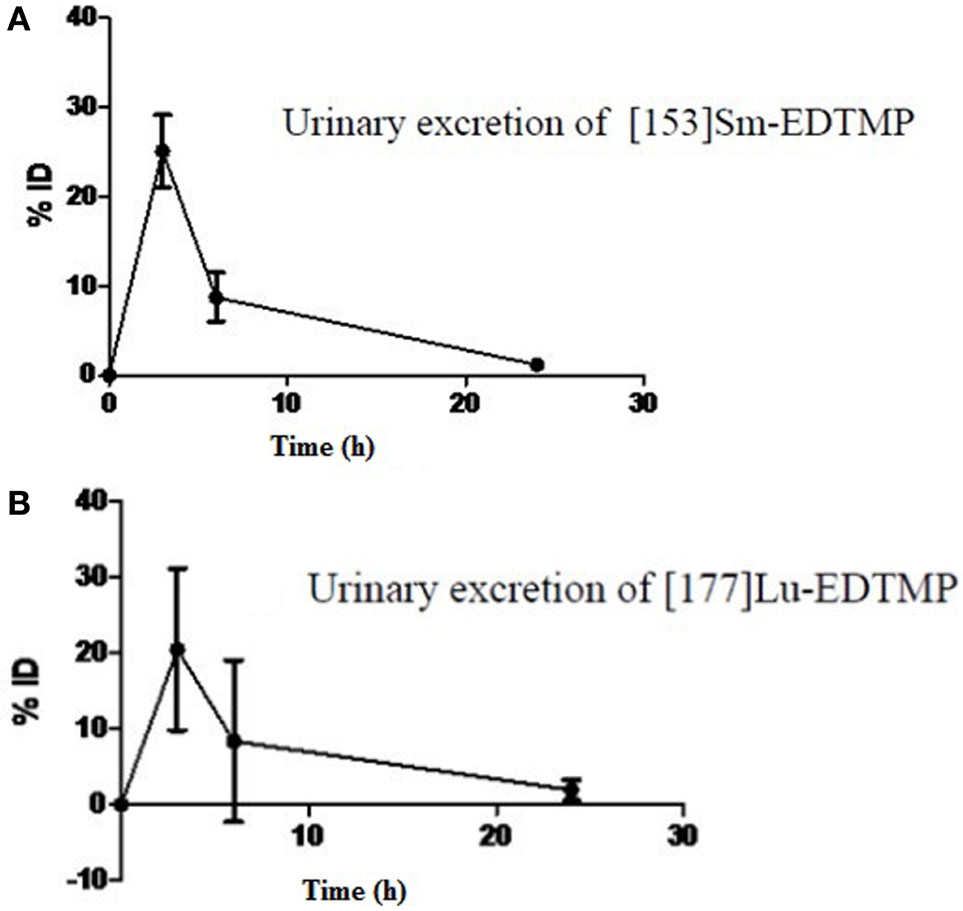
**Urinary excretion pattern of (A) ^153^Sm-EDTMP (B) ^177^Lu-EDTMP**.

Following intravenous administration, both ^153^Sm-EDTMP and ^177^Lu-EDTMP cleared rapidly from blood with less than 5.0% of the injected dose remaining in the blood by 6 h (Figures 1A,B and 2A,B). About 20.0–25.0% of the administered activity of both the radiopharmaceuticals excreted in the urine within the first 3 h. A negligible amount of the activity was observed thereafter over the next 24 h (Figures 3A,B).

**Figure 4:**
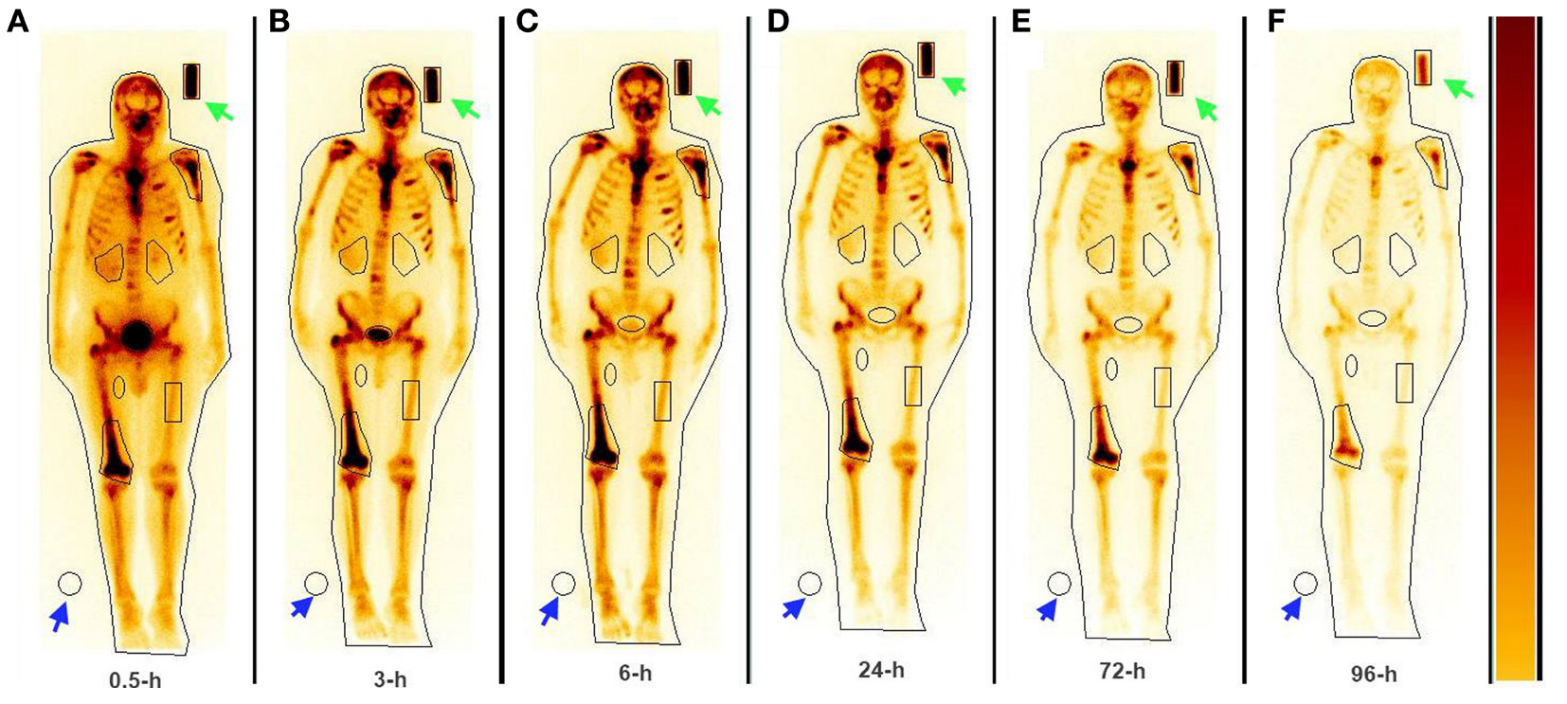
**The whole body anterior images acquired using a dual head gamma camera at 0.5, 3, 6 24, 72, and 96 h (A–F) following intravenous administration of ^153^Sm-EDTMP demonstrating intense localization of the tracer in multiple bone metastatic sites**.

**Figure 5:**
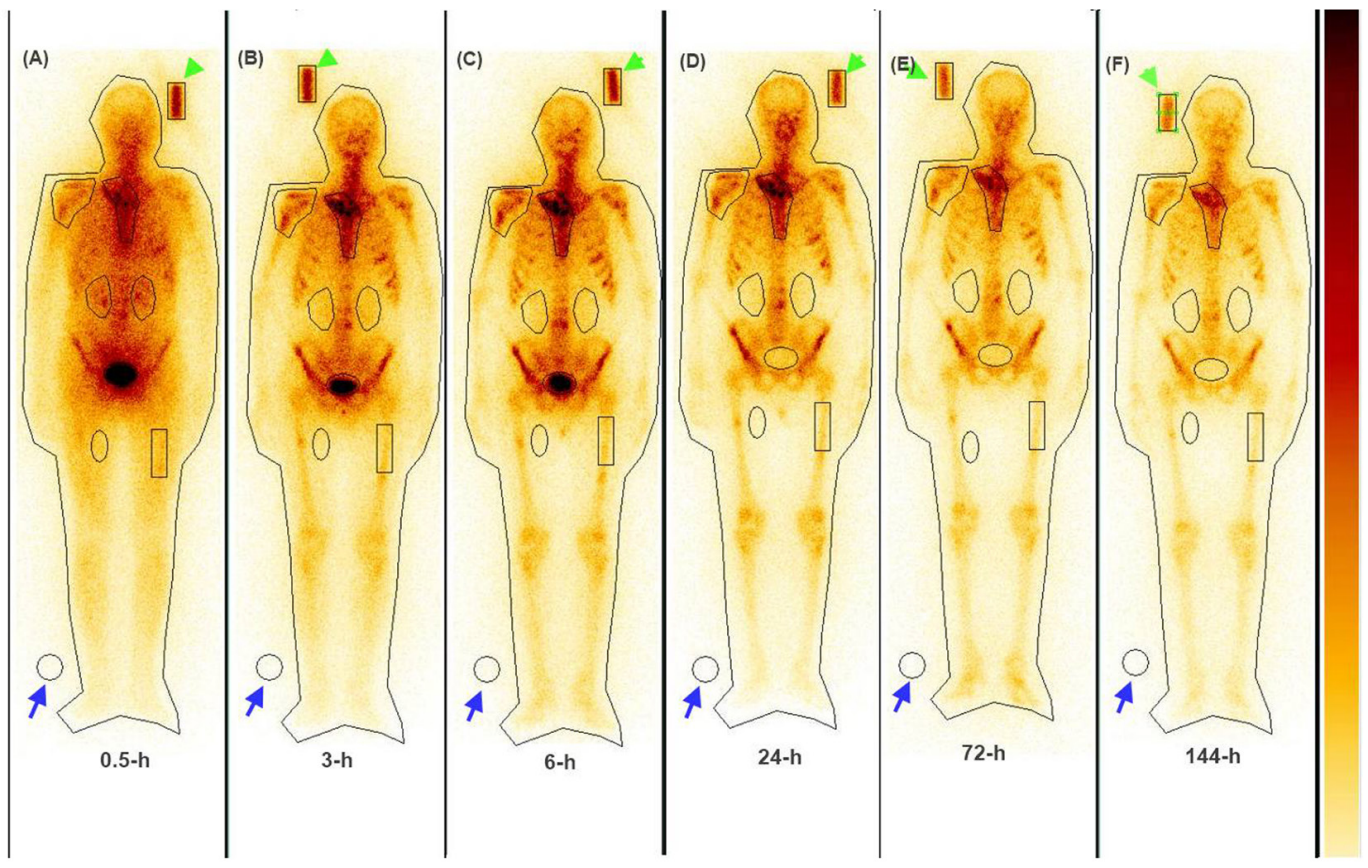
**The whole body anterior images acquired using a dual head gamma camera at 0.5, 3, 6, 24, 72, and 144 h (A–F) following intravenous administration of ^177^Lu-EDTMP demonstrating intense localization of the tracer in bone metastatic sites**.

The serial imaging data demonstrated that both ^153^Sm-EDTMP and ^177^Lu-EDTMP exhibited a rapid blood and soft tissue clearance. The skeletal metastatic lesions showed increased uptake of the radiopharmaceuticals which remained consistent throughout the imaging sequences (Figures 4 and 5, anterior images). A total of 86 (53 on ^153^Sm-EDTMP and 33 on ^177^Lu-EDTMP images) metastatic lesions were evaluated for absorbed dose calculations. Most common metastatic sites were observed in sternum, vertebrae, shoulder joint, femur, and ribs. However, in one patient, skull bone was also involved. The mean absorbed doses received by the metastatic sites were 6.22 ± 4.21 and 6.92 ± 3.92 mSv/MBq in ^153^Sm-EDTMP and ^177^Lu-EDTMP treated patients, respectively.

The results for human absorbed dose calculations in various organs following administration of ^153^Sm-EDTMP and ^177^Lu-EDTMP are presented in Table 3. The mean absorbed dose to bone from ^177^Lu-EDTMP was observed to be 5.26 ± 1.40 mSv/MBq which was slightly higher than (4.04 ± 2.47 mSv/MBq) that observed from ^153^Sm-EDTMP. However, the difference was not significant. The mean absorbed dose to kidneys from ^153^Sm-EDTMP treatment was 0.124 ± 0.20 mSv/MBq which was significantly higher (*p* < 0.001) than that observed (0.06 ± 0.04 mSv/MBq) with ^177^Lu-EDTMP treatment. On the contrary, the mean absorbed dose to the urinary bladder was significantly (*p* < 0.001) higher (1.35 ± 1.05 mSv/MBq) in ^177^Lu-EDTMP treated patients than (0.64 ± 0.34 mSv/MBq) in ^153^Sm-EDTMP treated patients. The mean absorbed dose to testes in ^177^Lu-EDTMP patients was significantly (*p* < 0.001) lower (0.05 ± 0.04 mSv/MBq) as compared with ^153^Sm-EDTMP patients. The statistical analysis using two tailed unpaired “*t*” test indicated that no significant (*p* > 0.05) difference was observed in the mean absorbed dose, total body dose, effective dose to various organs in the two groups of patients treated with ^153^Sm-EDTMP and ^177^Lu-EDTMP.

**Table 3:** **The mean absorbed organ dose, total body, and effective dose received (mSv/MBq) in ^153^Sm-EDTMP- and ^177^Lu-EDTMP-treated patients**.

	Absorbed dose in mSv/MBq			
	^153^Sm-EDTMP		^177^Lu-EDTMP	
Target organ	Mean	SD	Mean	SD
Adrenals	0.069	0.059	0.058	0.039
Brain	0.077	0.079	0.058	0.038
Breasts	0.070	0.052	0.064	0.055
Gallbladder wall	0.075	0.055	0.052	0.041
LLI wall	0.076	0.057	0.096	0.088
Small intestine	0.065	0.062	0.060	0.047
Stomach wall	0.073	0.058	0.050	0.040
ULI wall	0.065	0.052	0.053	0.040
Heart wall	0.098	0.090	0.054	0.040
Kidneys	0.124	0.201	0.060	0.042
Liver	0.068	0.058	0.072	0.057
Lungs	0.076	0.055	0.054	0.039
Muscle	0.051	0.046	0.066	0.045
Ovaries	0.066	0.060	0.057	0.039
Pancreas	0.078	0.056	0.054	0.040
Red marrow	**1.413**	**0.607**	**0.833**	**0.213**
Osteogenic cells	**4.037**	**2.471**	**5.255**	**1.404**
Skin	0.067	0.053	0.058	0.042
Spleen	0.063	0.062	0.062	0.043
Testes	0.166	0.296	0.052	0.039
Thymus	0.066	0.059	0.050	0.040
Thyroid	0.070	0.054	0.053	0.039
Urinary bladder wall	**0.644**	**0.334**	**1.356**	**1.051**
Uterus	0.075	0.054	0.059	0.037
Total body	0.095	0.079	0.194	0.077
Effective dose equivalent	0.194	0.231	0.456	0.152
Effective dose	0.482	0.296	0.264	0.037

*Bold values demonstrate the significant changes*.

### Pain Assessment: Pain Scoring

In ^153^Sm-EDTMP-treated patients, 16 (16/20) patients were responders and the remaining 4 patients were non-responders. In responders, the mean pain score values were 5.75 ± 0.7, 4.31 ± 0.09, 2.4 ± 0.51, and 1.31 ± 0.5 at 1, 3, 6, and 8 weeks after the treatment (Table 4). The mean pain score in responders declined to 1.31 ± 0.48 at 8 weeks from the baseline score of 7.2 ± 1.72. Likewise, in ^177^Lu-EDTMP patients, 8 (8/10) were responders and the remaining 2/10 were non-responders. In responders, the mean pain score values were 5.25 ± 1.04, 4.38 ± 1.06, 2.63 ± 0.52, and 1.63 ± 0.5 at 1, 3, 6, and 8 weeks after the treatment. The mean pain score in responders declined to 1.63 ± 0.52 at 8 weeks from the baseline score of 7.9 ± 1.55. The statistical analysis of the pain score data demonstrated that a significant (*p* < 0.0001) decrease in pain score was noted at each post-therapy (with both the radiopharmaceuticals) assessment period when compared with the baseline value.

**Table 4:** **Mean pain scores of the patients over 8 weeks following treatment**.

Time (weeks)	Pain score (*p* value) of ^153^Sm-EDTMP	Pain score (*p* value) of ^177^Lu-EDTMP
0	7.19 ± 1.72	7.88 ± 1.55
1	5.75 ± 0.68*	5.25 ± 1.04*
3	4.31 ± 0.87*	4.38 ± 1.06*
6	2.44 ± 0.51*	2.63 ± 0.52*
8	1.31 ± 0.48*	1.63 ± 0.52*

*Indicates p value of <0.0001 at 95% confidence interval.

The response rate for each radionuclide in terms of a significant reduction in pain score was evaluated as about 80.0%. A statistical analysis using chi-square test did not show any statistical significant association (χ^2^ = 1 and *p* = 0.317) in pain relief among responders and non-responders in these two group of patients. No statistically significant correlation was observed between the absorbed dose to the metastatic sites and pain score.

### Toxicity Assessment and Survival Analysis

**Figure 6:**
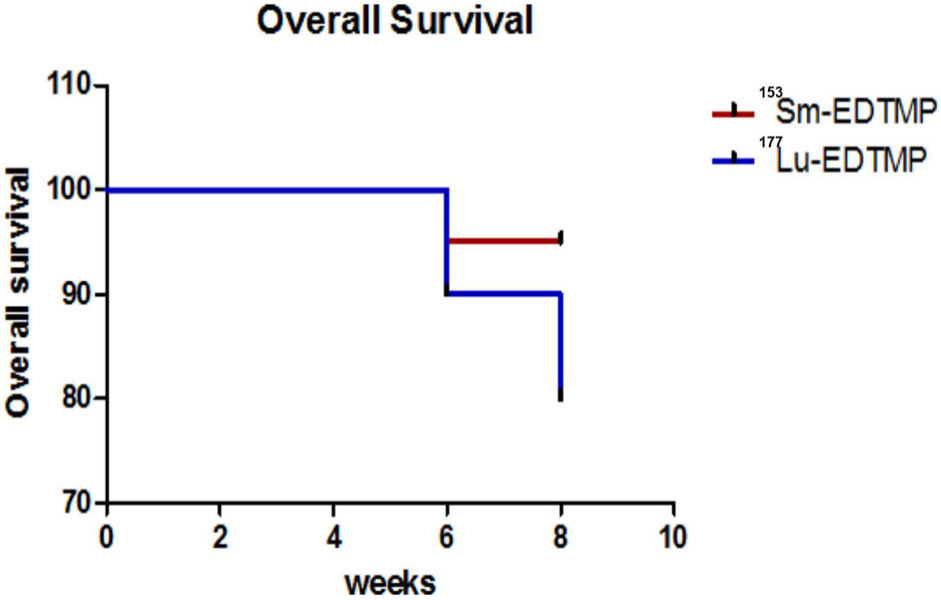
**Kaplan–Meier survival curve in patients treated with ^153^Sm-EDTMP and ^177^Lu-EDTMP**.

A mild to severe toxicity was observed in one patient each treated with ^153^Sm-EDTMP and ^177^Lu-EDTMP, respectively. These findings suggest that either of the two therapeutic radiopharmaceuticals can be used safely and confidently for achieving comparable therapeutic efficacy. Twenty-seven (27/30) patients survived for up to the end of 8 weeks of the study period with a significant pain reduction. Two patients (treated with ^177^Lu-EDTMP) died after eighth week and one patient (treated with ^153^Sm-EDTMP) at 4 weeks of treatment, respectively. A Kaplan–Meier survival analysis curve of patients treated with ^153^Sm-EDTMP and ^177^Lu-EDTMP is presented in Figure 6.

## Discussion

An irradiation of the bone metastatic lesions with minimal radiation effect on the surrounding normal tissue with the use of short tissue range beta emitters provides a significant bone pain palliation in patients with multiple skeletal metastases. In the present study, we treated 20 patients with ^153^Sm-EDTMP and 10 patients with ^177^Lu-EDTMP using a fixed dose protocol of 37.0 MBq/kg body weight of each of the two radiopharmaceuticals. The bone metastatic lesions seen on bone scanning were matched with the corresponding lesions on serial ^153^Sm-EDTMP and ^177^Lu-EDTMP images. The accurate mapping is necessary to ensure a significant dose delivery to the bony metastatic sites to achieve the predicted therapeutic outcome of RN therapy in these patients (28).

The comparative dosimetry data analysis revealed that the mean absorbed doses to the bony metastatic lesions in ^153^Sm-EDTMP- and ^177^Lu-EDTMP-treated patients were 6.22 ± 4.21 and 6.92 ± 3.92 mGy/MBq, respectively. These values were not significantly different from each other. We could not find the reference absorbed dose data to the metastatic lesions in context with either ^153^Sm-EDTMP or ^177^Lu-EDTMP. However, the mean absorbed dose values to the metastatic lesions have been reported for ^186^Re-HEDP and ^188^Re-HEDP (29, 30) previously. These authors reported that the median and mean absorbed dose values were 26.0 and 12.4 ± 6.2 Gy for ^186^Re-HEDP (85.0 mCi) and ^188^Re-HEDP, respectively. In our study, an administration of mean dose of 2220 MBq either of ^153^Sm-EDTMP and ^177^Lu-EDTMP to a patient will deliver a mean absorbed dose of about 14.0 Gy to the bone metastases which is comparable with the absorbed dose estimates with ^186^Re-HEDP or ^188^Re-HEDP as reported by these authors. These results therefore suggest an adequate cumulative absorbed dose delivery to the metastatic lesions for sustained pain relief palliation effect over an extended period.

Further, in the present study, both the radiopharmaceuticals, i.e., ^153^Sm-EDTMP and ^177^Lu-EDTMP exhibited a similar response (bone pain reduction) rate (80.0%) and pain-free survival period. Our results for ^153^Sm-EDTMP are in agreement with the previous studies which have shown response rate ranging between 65 and 86% (21, 31, 32).

In a recent study, Shinto et al. (33) used the same indigenous production ^177^Lu-EDTMP at a fixed dose of 3,700.0 MBq and reported a complete pain relief at 12 weeks in their patients. Further, Yuan et al. (34) reported that a fixed dose of 2,590 MBq of ^177^Lu-EDTMP exhibited a response rate of 80.0%. However, these authors reported a lower response rate of 55.0% while using a lower dose of 1,295 MBq as a part of their comparative analysis. Recently, Agarwal et al. (35) in a group of 44 patients treated with ^177^Lu-EDTMP reported an overall response rate of 86%. They further observed that complete, partial, and minimal response rate was seen in 13, 48, and 25% patients, respectively. These results are comparable with ^89^SrCl_2_ which has been the most extensively used radiopharmaceuticals for bone pain palliation especially in the western countries. However, ^89^SrCl_2_ has to be imported at an exorbitant cost and has been reported to cause a significant myelotoxicity (36). Therefore, we need to use the indigenously developed therapeutic agents with least myelotoxicity and which can be made available to the patients at an affordable cost in our country.

We observed that both the indigenously produced radiopharmaceuticals are very safe for human administration with observation of no untoward incidence, pain flare, or change in hematological parameters. However, one patient each in the two groups of patients treated with ^153^Sm-EDTMP and ^177^Lu-EDTMP developed grade III/IV and I/II toxicity, respectively. There are varied reports on toxicity with the use of these therapeutic radionuclides (21, 32). However, Shinto et al. reported no incidence of any toxicity in their preliminary study using 3,700.0 MBq dose of ^177^Lu-EDTMP (33).

The results of our study demonstrates that ^177^Lu-EDTMP delivers lesser (0.83 ± 0.21 mSv/MBq) red marrow absorbed dose than (1.41 ± 0.61 mSv/MBq) that observed with ^153^Sm-EDTMP treatment. The mean absorbed dose to the lesions following RN therapy with both ^153^Sm-EDTMP and ^177^Lu-EDTMP was similar and was six to seven times higher than the bone marrow absorbed dose and thereby offering high target to non-target ratio. The bone marrow absorbed dose from ^177^Lu-EDTMP treatment in a recently published Indian study has been reported to be 0.8 mGy/MBq, which is similar to our results (37). Therefore, ^177^Lu-EDTMP seems to be a promising alternative for bone pain palliation therapy. The bone marrow absorbed dose from ^153^Sm-EDTMP treatment in previous studies has been reported to be ranging between 0.89 and 1.86 mGy/MBq (38–40). This difference in bone marrow absorbed dose could be due to the lower beta energy of ^177^Lu as compared to that of ^153^Sm. However, it has been reported that the lower β-energy of ^177^Lu and relatively longer half-life will allow the deposition of an adequate tumor irradiation dose at a constant rate (41). However, the mean absorbed doses to the bone (target organ) following ^153^Sm-EDTMP (4.04 ± 2.47 mSv/MBq) and ^177^Lu-EDTMP (5.26 ± 1.40 mSv/MBq) were comparable.

In the present study, the mean absorbed radiation doses to the kidneys and urinary bladder were 0.124 ± 0.20 and 0.64 ± 0.34 mSv/MBq and 0.06 ± 0.04 and 1.35 ± 1.05 mSv/MBq, respectively, in ^153^Sm and ^177^Lu-treated patients. With the use of either of these two radiopharmaceuticals, the kidney and bladder absorbed doses are well below the maximum permissible dose limits of 23.0 and 2.0 Gy, respectively.

Both ^153^Sm-EDTMP and ^177^Lu-EDTMP offered good image quality for performing individualized patients’ dosimetry. The statistical analysis indicated that no significant (*p* > 0.05) difference was observed in the mean absorbed dose, total body dose, effective dose to various lesions/organs in patients treated with ^153^Sm-EDTMP and ^177^Lu-EDTMP. These results indicated that both the radionuclides have the similar normal human biodistribution and deliver the same radiation dose to the various metastatic lesions and body organs and therefore can be used interchangeably depending upon the availability in a given setting.

The present study thus highlights that both ^177^Lu-EDTMP and ^153^Sm-EDTMP provide competitive therapeutic efficacy for achieving bone pain palliation, but the same needs to be established in a large number of cancer patients through multi-centric trials prospectively.

## Ethics Statement

This study was carried out in accordance with the recommendations of “the guidelines of the Institute Ethics committee”-IEC with written informed consent from all subjects. All subjects gave written informed consent in accordance with the Declaration of Helsinki. The protocol was approved by the “IEC and IBC.”

## Author Contributions

Study design, experimental work, imaging and data analysis, and manuscript writing: all authors.

## Conflict of Interest Statement

The authors declare that the research was conducted in the absence of any commercial or financial relationships that could be construed as a potential conflict of interest. The reviewer, MD, and handling editor declared their shared affiliation, and the handling editor states that the process nevertheless met the standards of a fair and objective review.
